# A generalized protocol for the induction of M2-like macrophages from mouse and rat bone marrow mononuclear cells

**DOI:** 10.1093/biomethods/bpaf020

**Published:** 2025-03-19

**Authors:** Ulugbek R Yakhshimurodov, Kizuku Yamashita, Kenji Miki, Takuji Kawamura, Shunsuke Saito, Shigeru Miyagawa

**Affiliations:** Department of Cardiovascular Surgery, Graduate School of Medicine, Osaka University, Suita, 565-0871, Japan; Department of Cardiovascular Surgery, Graduate School of Medicine, Osaka University, Suita, 565-0871, Japan; Premium Research Institute for Human Metaverse Medicine (PRIMe), Osaka University, Suita, 565-0871, Japan; Department of Cardiovascular Surgery, Graduate School of Medicine, Osaka University, Suita, 565-0871, Japan; Department of Cardiovascular Surgery, Graduate School of Medicine, Osaka University, Suita, 565-0871, Japan; Department of Cardiovascular Surgery, Graduate School of Medicine, Osaka University, Suita, 565-0871, Japan

**Keywords:** mouse, rat, M2, macrophage, protocol

## Abstract

Regardless of origin and localization, macrophages are the major immune cells that maintain homeostasis in both healthy and diseased states. However, there is no consensus on the phenotypes, functions and fates of macrophages. Existing studies clarify macrophage biology from different biomedical research perspectives, but the heterogeneity of induction methods hinders reproducibility and comparability. To address this problem, we validated a novel generalized *in vitro* protocol for the induction of M2-like macrophages from mice and rats bone marrow mononuclear cells. Our approach improves reliability and cross-species applicability, providing a valuable tool for macrophage research.

## Introduction

Macrophages, present in every organ of the body, are key players in the immune system as professional phagocytes whose role was first recognized by Metchnikoff [1]. Despite their common fundamental functions, macrophages exhibit considerable heterogeneity in terms of their origin, location, gene expression, response to stimuli and downstream functions [2]. Traditionally, macrophages have been classified into proinflammatory (M1) and anti-inflammatory (M2) phenotypes on the basis of cytokine and chemokine profiles [1,3]. However, genomic studies suggest minimal differences between these subtypes, highlighting the complexity of macrophage heterogeneity and its active exploration in current research [4]. Earlier studies classified macrophages into classically activated (proinflammatory), alternatively activated (anti-inflammatory) and type II activated types, each of which is associated with different biological functions [3]. But newer models recognize a wider spectrum of activation states, including naive macrophages (M0), proinflammatory (M1), various anti-inflammatory subtypes (M2a, M2b, M2c, M2d) and intermediate phenotypes, such as Mox or M4 [5]. This evolving understanding emphasizes the subtle and dynamic role of macrophages in immunity and tissue homeostasis. Given the importance of macrophages, it is evident that the fine control of macrophage plasticity in the transition from one subtype to another determines not only the magnitude but also the nature of the immune response and disease outcome, as shown in recent studies elucidating their potential therapeutic efficacy and safety [6–13].

A variety of cells and/or progenitors are utilized to differentiate anti-inflammatory macrophages *in vitro*, including embryonic stem cells [14–17], peripheral blood mononuclear cells [17], induced pluripotent stem cells [18,19], and certainly bone marrow mononuclear cells [20–23] (BMNCs) stimulated with colony-stimulating factors and cytokines. Most of the existing protocols for macrophage differentiation rely on treatment with macrophage colony-stimulating factor (Mcsf), often followed by stimulation with interleukin-4 (Il4), Il10, and Il13 to enhance anti-inflammatory properties. However, there are several limitations in those protocols that should be considered: (i) variability and reproducibility issues, where differentiation efficiency heavily depends on cell sources, cytokine concentrations, and culture conditions which hinders standardization; (ii) time, manpower, and obviously, cost constraints; (iii) complexity — multi-step processing and sequential stimulation, increasing the risk of batch-to-batch variation; (iv) species-specific differences — mammalian macrophages, particularly those derived from mouse and rat, exhibit distinct gene expression patterns, cytokine responses, and functional properties, complicating the development of a universally applicable protocol; and finally (v) ethical concerns — the necessity to use large numbers of animals contradicts the 3R principles (Replace, Reduce, Refine), emphasizing the need for an optimized, efficient, reproducible, and humane alternatives.

**Figure 1:**
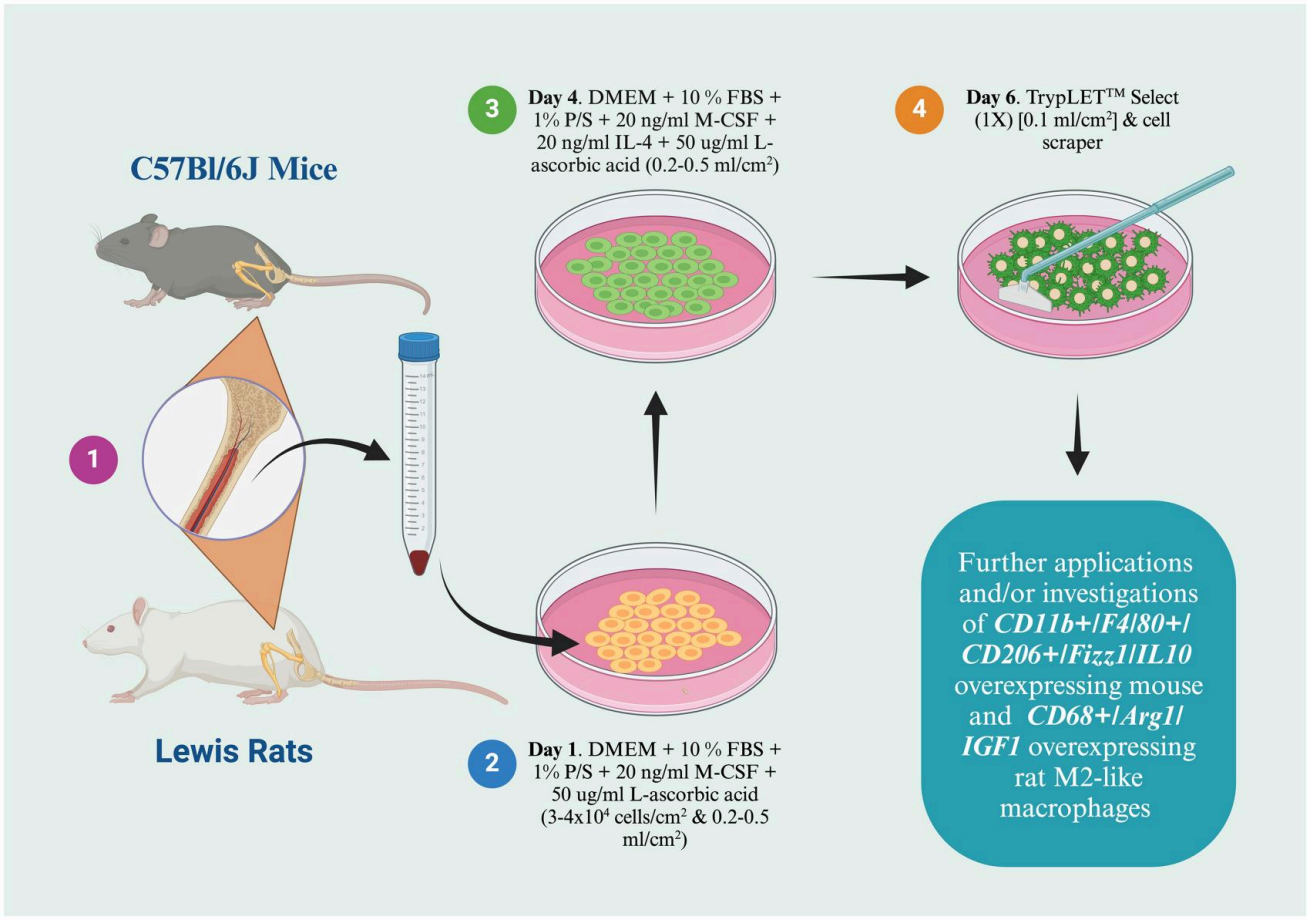
Schematic overview of the protocol for the induction of M2-like macrophages. After isolation under sterile conditions, long tubular bones were washed with cold complete DMEM, and BMNCs were seeded in Cocktail 1 (0.2–0.5 ml/cm^2^ and 3–4×10^4^ cells/cm^2^) for 72 h or 3 days. On day 4, the medium was replaced with Cocktail 2 (0.2–0.5 ml/cm^2^) by aspirating the old medium and adding new medium. After 48 h of medium change, induced M2-like macrophages were harvested and used for further investigations. *BMNCs—bone marrow mononuclear cells; FBS—fetal bovine serum; P/S—penicillin/streptomycin;* Created in BioRender. https://BioRender.com/c16h782

To address these challenges, we validated a standardized and efficient protocol for inducing M2-like macrophages from mouse and rat BMNCs (Fig. 1). This method is high-throughput, time-efficient, operator-independent, and compliant with 3R principles, providing a valuable tool for immunology studies. To the best of our knowledge, this is the first study describing a generalized approach that provides reproducible results for both species (mice and rats), thus increasing the translational potential of macrophage studies.

## Research highlights

1. This protocol is easy to perform, and less time and expertise are needed.
2. Since the total induction time is shorter and only recombinant mouse/rat Mcsf is used in the first 72 h of induction, an even smaller budget can be considered.
3. The cell yield is as high as 200%, making this protocol more efficient.
4. To the best of our knowledge, this is the first protocol describing a generalized method to obtain M2-like macrophages from mouse and rat BMNCs.

## Materials and methods

### Animals

All the animal studies were approved by the Institutional Animal Care and Use Committee (IACUC) of Osaka University, Japan. Experiments were performed on pathogen-free 6–8-week-old male Lewis rats (Japan SLC, Inc.) and 8–10-week-old male C57Bl/6J mice (The Jackson Laboratory). The animals were housed in facilities at Osaka University Graduate School of Medicine in an air-conditioned room with a 12-h light–dark cycle and free access to food and drink.

### Reagents and equipment

A complete list of reagents used in this study is given in Table 1.

**Table 1.** List of reagents used in this study.

TaqMan probes		
Gene	Manufacturer	Assay ID
*Gapdh*	Thermo Fisher	Rn01775763_g1
*Igf1*		Rn00710306_m1
*Arg1*		Rn00691090_m1
*Il1b*		Rn00580432_m1
*Il6*		Rn01410330_m1
*Il10*		Rn00563409_m1
*Nos2*		Rn00561646_m1
*Vegfa*		Rn01511601_m1
*Gapdh*		Mm99999915_g1
*Tgfb1*		Mm01178820_m1
*Il10*		Mm00439614_m1
*Tnf*		Mm00443258_m1
*Retnla*		Mm00445109_m1
*Hgf*		Mm01135193_m1
*Vegfa*		Mm01281449_m1
*Mmp2*		Mm00439498_m1

Proteins and antibodies		
Item	Manufacturer	Catalog number
Recombinant Rat Mcsf	Peprotech	400-28
Recombinant Rat Il4	Peprotech	400-04
Recombinant Murine Mcsf	Peprotech	315-02
Recombinant Murine Il4	Peprotech	214-14
Mouse anti rat CD68: Alexa Fluor^®^ 647	Bio-Rad	MCA341A647
Mouse IgG1 negative control: Alexa Fluor^®^ 647	Bio-Rad	MCA1209A647
BD™ CompBead Plus Anti-Rat Ig, κ/Negative Control (BSA) Compensation Plus (7.5 μm) Particles Set	BD Bioscience	560499
Fixable Viability Stain 780	BD Bioscience	565388
BD™ CompBead Plus Anti-Mouse Ig, κ/Negative Control (BSA) Compensation Plus (7.5 µm) Particles Set	BD Bioscience	560497
Anti-CD16+CD32 antibody [93]—BSA and Azide free	Abcam	ab25235
BD Pharmingen^TM^ DAPI Solution	BD Pharmingen	564907
APC Anti-CD11b antibody [M1/70]	Abcam	ab25482
APC Mouse IgG2b, κ Isotype Ctrl Antibody	Bio Legend	400322
Alexa Flour^®^ 488 anti-mouse CD206 (MMR) Antibody	Bio Legend	141710
Alexa Flour^®^ 488 Rat IgG2a, κ Isotype Ctrl Antibody	Bio Legend	400525
F4/80 Monoclonal Antibody (BM8), PE, eBioscience^TM^	Thermo Fisher	12-4801-82
Rat IgG2a, κ Isotype Ctrl (eBR2a), PE, eBioscience	Thermo Fisher	12-4321-42

**Buffers, medium, supplements, and others**		

L-ascorbic acid, powder, suitable for cell culture, γ-irradiated	Sigma‒Aldrich	A4403
DMEM (4.5 g/l Glucose) with L-Gln and Sodium Pyruvate, liquid	Nacalai Tesque	08458-45
Lysing Buffer	BD Bioscience	555899
D-PBS(−) without Ca and Mg, liquid	Nacalai Tescue	14249-95
Histopaque 1083	Sigma Aldrich	10831
Gibco^TM^ TrypLE^TM^ Select Enzyme (1×), no phenol red	Gibco	50-591-420
Leucoperm	Bio-Rad	BUF09
Stain Buffer (FBS)	BD Bioscience	554656
Stain Buffer (BSA)	BD Bioscience	554657

*Note:* Cell culture dishes can be chosen depending on the scale, purpose and budget of the experiments, so we do not recommend any particular type or manufacturer here.

### Bone excision (femurs and tibiae)

Isolation of long tubular bones has been described in detail in previous protocols and can therefore be found elsewhere. Briefly, the steps below should be followed.

Preparation: Sterile surgical tools, 70% ethanol, cold PBS (cPBS), high-glucose DMEM with 10% FBS and 1% P/S, sterile gloves, and waste disposal materials.

*Procedure (20–30 min):*

1. Euthanize the mouse/rat following institutional guidelines.
2. Shave the lower body and wipe with paper to remove contaminants.
3. Immerse the body in 70% ethanol for 5 min, place on sterile gloves.
4. Secure the body in the supine position, cover unshaven areas with sterile gauze, and immobilize the limbs with sterile needles.
5. Make a precise skin incision from the ankle to the hip joint, exposing and detaching the femur and tibia.
6. Remove soft tissue using sterile forceps and scissors.
7. Place the bones in 70% ethanol (5 min) → cPBS (5 min) → cold DMEM (5 min) to ensure sterility.
8. Store bones on ice or at 4°C if immediate processing is not possible.
*Note: Short-term processing in 70% ethanol will not damage BMNCs if bone integrity is guaranteed. Avoid working with fractured bones to prevent contamination and cell loss. If an aseptic environment is ensured, the* ***ethanol*** *step may be omitted.*

### BMNCs isolation

Preparation: Sterile surgical tools, cell strainers (70 μm), scrapers, syringes (5–10 ml), 22G/24G needles, 15–50 ml tubes, cell culture dishes, DMEM, PBS, Histopaque-1083 placed at room tempurature (RT) for at least 30 min before use, centrifuges, trypan blue, cell counters, and waste disposal materials.

*Procedure (60–90 min):*

**Figure 2:**
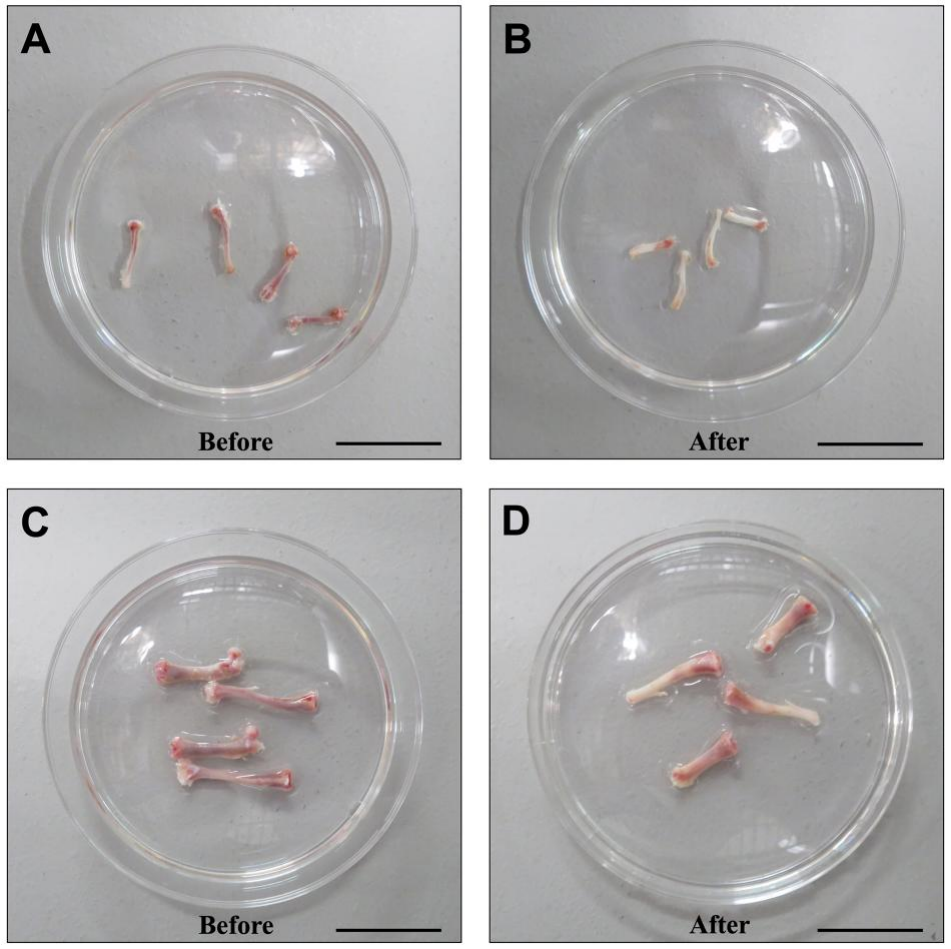
Femur and tibia bones isolated from mice (A, B) and rats (C, D) before and after the flushing process. Great care must be taken when processing bones, as there is a risk of fracture and contamination. Scale bars are 3 cm

1. Transfer bones from DMEM to a Petri dish with cPBS, using two dishes for sequential cleaning.
2. Remove residual muscle/tendon with a disposable scalpel.
3. Cut off the bone epiphyses, keeping the shafts as long as possible (*the longer the shafts, the more cells)* to allow access to the bone marrow (BM) using 24 and 22 gauge needles in mice and rats, respectively, and puncture both ends of the bone shaft.
4. Flush BM into a sterile 50 ml tube with DMEM (2–3 ml per bone for mice, 4–5 ml for rats). If the bones are washed correctly, the color of the middle part should change from pink or pale red to yellow (Fig. 2).
5. Filter BM suspension through a 70 μm cell strainer, using a scraper to dislodge solid marrow.
6. Centrifuge at 1200 rpm, 4°C, 5 min; discard supernatant.
7. Resuspend pellet in 1 ml PBS, then add another 4 ml (6 ml for rats), pipette up and down for homogenization.
8. Carefully layer the cell suspension onto an equal volume of Histopaque in a 15 ml tube (use 7 ml per tube for rats).
9. Centrifuge at 400 g, RT, 30 min with the lowest acceleration/deceleration settings.
10. Carefully remove and discard the upper PBS layer. Collect the opaque BMNCs layer into a fresh 15 ml tube.
11. Wash BMNCs by adding 10 ml PBS, and then centrifuging at 1200 rpm, RT, 5 min.
12. Discard supernatant and resuspend pellet in 1 ml pre-warmed DMEM, then add another 4 ml and pipette to mix.
13. Count cells using a hemocytometer or automated counter with trypan blue staining to assess viability.
*Note: To maximize yield, perform bone and BMNCs isolation on the same day, avoiding cold Histopaque to prevent clumping. If possible, the above steps should be performed in sterile areas or in safety cabinets to prevent contamination.*

### M2-like macrophage induction

Preparation: sterile gloves, cell culture dishes, DMEM, mouse/rat recombinant macrophage colony stimulating factor (Mcsf), L-ascorbic acid, and an incubator.

*Procedure: 15–20 min.*

1. Add 5 ml cell suspension from *step 13* above to the appropriate volume of *Cocktail 1* *(DMEM + Mcsf + L-ascorbic acid)*, which should be calculated by multiplying the surface area of the culture dish (e.g. 56.7 cm^2^ for a 100 mm dish) by 35 000 cells/cm^2^ and 0.2 ml/cm^2^ to determine the required number of cells and volume of medium per dish [24], respectively, ensuring that the final volume, when combined with the 5 ml cell suspension, must be equally divided into 12 ml portions, each containing approximately 2 million cells, as detailed in Table 2.
2. Mix well to evenly distribute the cells, cytokines and L-ascorbic acid in the medium.
3. Add 12 ml of *Cocktail 1* to each 100 mm dish, which then is labeled and incubated at 37°C (5% CO_2_ and 20% O_2_) for 72 h or 3 days.
*Note. It is recommended that gloves be changed, and the working area be cleaned before the cells are seeded. Depending on the scale of the experiments, further investigations, budget, actual need for M2-like macrophages, and total number of harvested BMNCs, various cell culture dishes ranging from 35 to 150 mm, as well as multiwell plates and flasks, could be used. The only thing to consider is the total surface area of the selected cell culture dish, as this is important for calculating the appropriate volume of medium (0.2–0.3 ml/cm^2^) and number of cells (3–4×10^4^ cells/cm^2^) recommended by the manufacturer [24]. In addition, a cell scraper can optionally be used to collect M2-like macrophages, which may also have an impact on the choice of cell culture dishes.*

**Table 2.** Composition of cell culture medium.

Reagents	Dose	Cell number (cells/cm^2^)	Medium (ml/cm^2^)	Cocktail 1	Cocktail 2
Recombinant Mcsf	20 ng/ml	3.5–4×10^4^	0.2–0.3	+	+
Recombinant Il4	20 ng/ml			−	+
L-ascorbic acid	50 µg/ml			+	+
DMEM (high glucose)	1 ml			+	+

### Medium change

Preparation: sterile gloves, laboratory coats, 50 ml tubes, DMEM, recombinant mouse/rat Mcsf, interleukin 4 (Il4), and L-ascorbic acid.

*Procedure: 10–15 min.*

1. On day 4 or 70–72 h after induction, aspirate and discard the existing medium.
2. Add the required volume (e.g. 12 ml per dish in the case of 100 mm cell culture dishes) of *Cocktail 2* *(DMEM + Mcsf + Il4 + L-ascorbic acid) to each culture dish*, as described in Table 2.
3. Incubate for another 48 h or 2 days at 37°C.
*Note. The Dulbecco’s modified Eagle’s medium (DMEM) should be pre-warmed before use.*

### M2-like macrophage harvesting

Preparation: sterile gloves, laboratory coats, cell scrapers, DMEM, cPBS (or alternative buffers based on downstream applications), slides for cell counting, trypan blue (or alternatives), trypsin, and sterile 15 ml tubes.

*Procedure: depending on the number and type of cell culture dishes, the duration can vary from 20 to 60 min or even longer.*

1. On day 6 or 118–124 h after induction, remove the medium by aspiration.
*Optional. The normal medium may be changed to serum-free medium 24 h before cell harvesting, and the next day medium can be collected in a sterile tube and used for protein-based assays.*
2. Add 5 ml of trypsin to each dish.
3. Incubate at 37°C for 2–3 min.
4. Check culture dishes under a light microscope to determine whether they had detached. In most cases, trypsinization is not strong enough to detach the cells, so mechanical intervention is necessary.
5. To minimize the cell loss, add the equal volume of complete DMEM (10% FBS and 1% P/S) onto trypsin and gently and thoroughly scrape the entire surface.
6. Shake the dish, pipette several times, and collect the cell suspension in a 15 ml tube. Rinse with 4–5 ml of cPBS to collect the remaining cells.
7. Centrifuge at 1200 rpm, 4°C, 5 min using the highest acceleration and deceleration settings.
8. Aspirate and discard the cell supernatant.
9. Add 1 ml of cPBS (or alternatives) and mix well with a pipette.
10. Count M2-like macrophages immediately or make a frozen stock in a cell banker (10^3^ cells/1 µl) at -80°C or -150°C.

## Macrophage differentiation assessment

### Microscopy

To assess changes in cell morphology (shape and size) and confluency, culture dishes were observed via an All-in-One Microscope BZ-X810 (Keyence Corporation, Osaka, Japan) at different time points: 24, 72, 96, and 120 h after induction. Images of M2-like macrophages were captured via differential interference contrast (DIC) microscopy at 20x magnification.

### mRNA extraction

The RLT buffer included in the RNeasy kit (Qiagen, Germany) and the RNase-Free DNase kit (Qiagen, Germany) were used to isolate RNA from M2-like macrophages as well as BMNCs (2*×*10^6^ cells/sample) and digest contaminating DNA, respectively, according to the manufacturer's protocol. The purified RNA concentration in the samples was measured, and the samples were then stored at −80°C for further applications.

### cDNA reverse transcription

A SuperScript VILO cDNA synthesis kit (Invitrogen) was used to produce first-strand cDNA for two-step real-time quantitative PCR (RT–qPCR) according to the manufacturer's instructions. Briefly, an appropriate volume containing 1 μg of RNA was combined with 4 μl of SuperScript VILO and RNase-free water, resulting in a final volume of 20 μl. All the obtained samples were lightly shaken by vortexing, spun and loaded into a thermocycler.

### RT–qPCR

PCR primers (Thermo Fisher Scientific) for the following genes were used in the assay of harvested cells: *Arg1, Fizz1, Tnfa, Il10, Igf1, Tgfb1, Hgf, Vegfa, Mmp2, Il1b, Il6,* and *iNos* (Table 1). BMNCs and *Gapdh* served as biological sample and internal control, respectively, with the latter aimed at normalizing the relative expression levels between samples. Briefly, 2.0 µl (10 ng) of cDNA was added to a 96-well MicroAmp^®^ Optical (Applied Biosystems) reaction plate with 10.0 µl of Thunderbird^®^ Next Probe qPCR Mix, 1.0 µl of TaqMan™ Assay, 2.0 µl of 1× ROX and 5.0 µl of nuclease-free water so that the total volume of one reaction was 20.0 µl. For each target gene, two technical replicates were performed. The RT–qPCR results were exported to MS Excel for further analysis, and relative quantification (RQ) values were used to compare gene expression levels between samples.

### Flow cytometry

There were significant differences between flow cytometry methods for mouse BMNCs-derived M2-like macrophages (mBMNCs-M2) and rat BMNCs-derived M2-like macrophages (rBMNCs-M2) due to the localization of phenotypic markers relative to the cell membrane, the availability and performance of conjugated antibodies, the variety of live/dead cell staining agents, etc. Briefly, cells collected in Section 2.7 were distributed into 5 ml flow cytometry tubes to obtain samples with 5*×*10^5^ cells/tube and washed once with cold (4°C) flow cytometry buffer (5% FBS, 0.002% NaN_3_ in PBS).

- mBMNCs-M2 were resuspended in 100 μl of flow cytometry buffer. The samples were blocked with an anti-mouse CD16/32 antibody (IgG2a, 93, monoclonal, rat; 1:100) and subsequently incubated with conjugated antibodies (Table 1) at 4°C for 30 min each, with protection from light. The cells were then washed once with flow cytometry buffer, resuspended in 500 μl of buffer and stained with DAPI (2 ng/μl) as a viability marker.
- rBMNCs-M2, unlike mouse macrophages, were resuspended in 0.5 ml of PBS for viability staining with 0.5 µl of BD Horizon™ Fixable Viability Stain 780 stock solution (1:1000) for 15 min at room temperature in a light-protected area. The cells were washed once with flow cytometry buffer, then fixed and permeabilized using Leucoperm (BUF09, Bio-Rad) to allow access to intracellular targets. They were incubated with a conjugated antibody against the pan-macrophage marker CD68 for 30 min at 4°C, followed by a final wash with flow cytometry buffer.

Before running the test, all the samples were transferred to flow cytometry tubes equipped with a filter cap. Unstained and matched IgG-stained samples served as negative controls and were used for calibration. The expression of extracellular and intracellular markers of macrophages was assessed via a BD Canto II flow cytometer. The obtained data were processed via BD FACSDiva Software v8.0.1/FlowJo v10, and 10 000 end-gated events were recorded for each sample. Appropriate compensation was performed before each experiment via compensation beads. Cell debris, doublets, and dead cells were excluded during the processing step.

## Quantification and data processing

### Cell output calculation

The total number of cells seeded on day 1 on each culture dish and harvested from each culture dish on day 6 were counted via trypan blue staining (0.4%, Nacalai Tesque) and automated cell counting (Countess© II Automated Cell Counter, Thermo Fisher). The cell output was calculated as follows:

$$\mathrm{Output}\;(\%)=\frac{\mathrm{Number}\;\mathrm{of}\;\mathrm{viable}\;\mathrm{harvested}\;\mathrm{cells}}{\mathrm{Number}\;\mathrm{of}\;\mathrm{viable}\;\mathrm{seeded}\;\mathrm{cells}}\;x\;100\%$$

### Statistical analysis

All the data are presented as the means±SEMs. Unpaired two-tailed Student's *t* test was used to analyze differences between experimental groups. All the statistical analyses were performed via GraphPad Prism software, version 10 (GraphPad Software, Inc., LA Jolla, CA, USA), and *P* < 0.05 was considered statistically significant.

## Results

### Il4 stimulates cell proliferation

**Figure 3:**
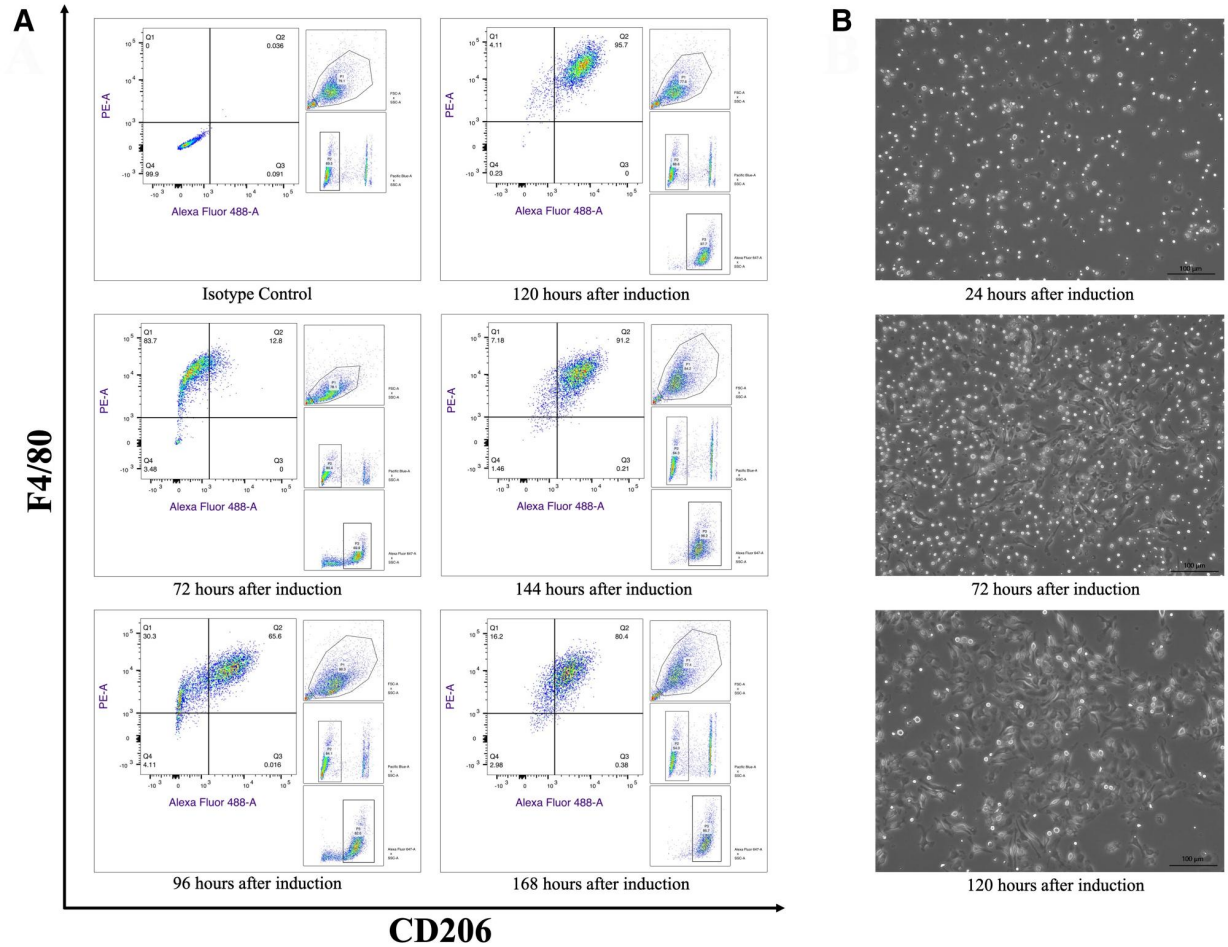
Phenotypic (A) and morphological (B) changes in mBMNCs-M2 over time. Most cells remained floating after 24 h of induction and even before the medium changed. Clear changes in cell shape and confluency were observed on days 5 and 6 under the influence of Il4. Phenotypic alterations coincided with morphological changes over time, as the number of F4/80 and CD206 positive cells increased dramatically, reaching 95.7% by day 6. Scale bars are 100 µm

**Figure 4:**
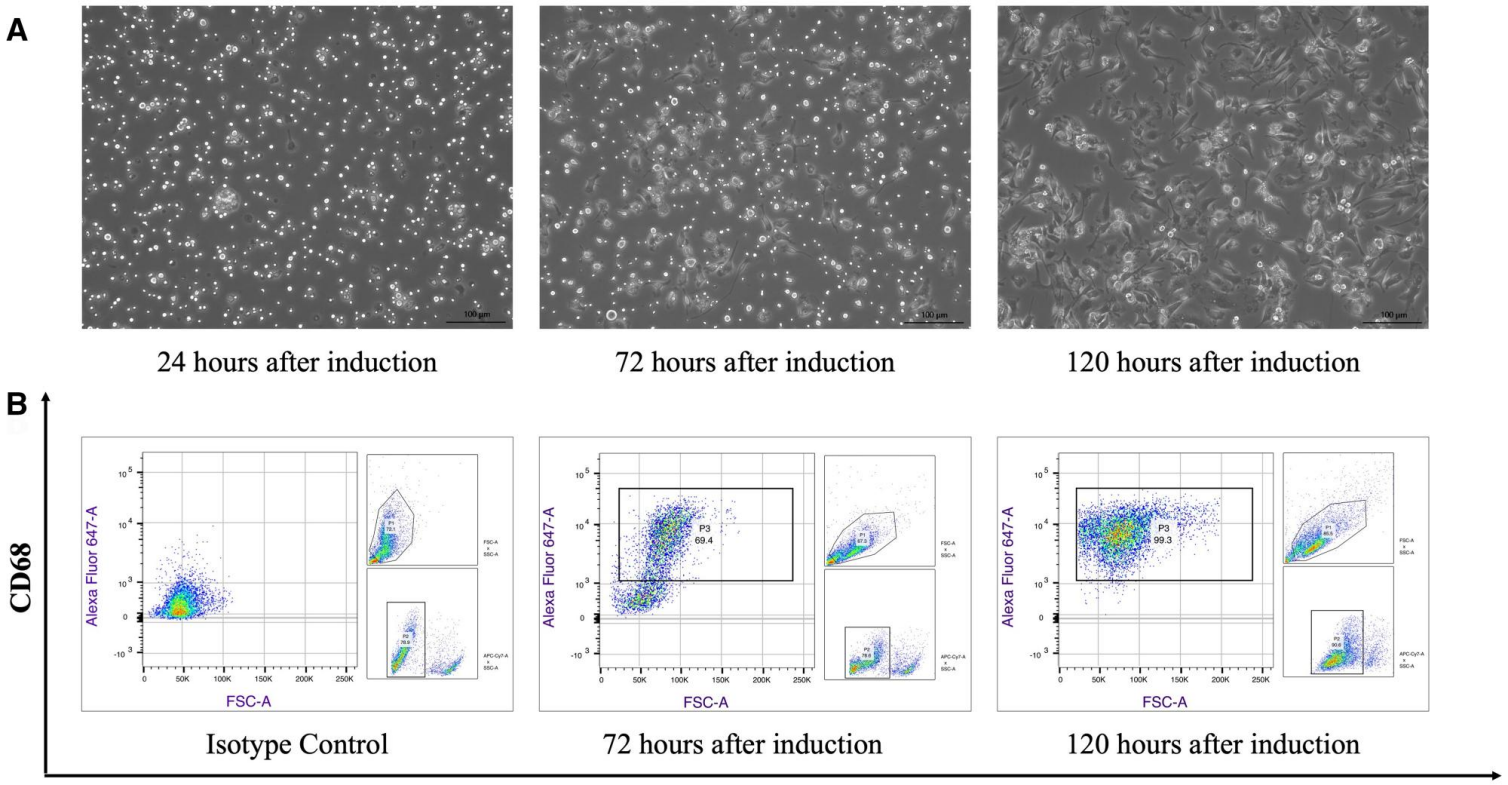
Morphological (A) and phenotypic (B) changes in rBMNCs-M2 over time. Significant alterations in cell shape were observed on days 5 and 6. The expression level of CD68, a marker of “panmacrophages,” increased over time to 69.4% and 99.3% on days 5 and 6, respectively. Scale bars are 100 μm

**Figure 5:**
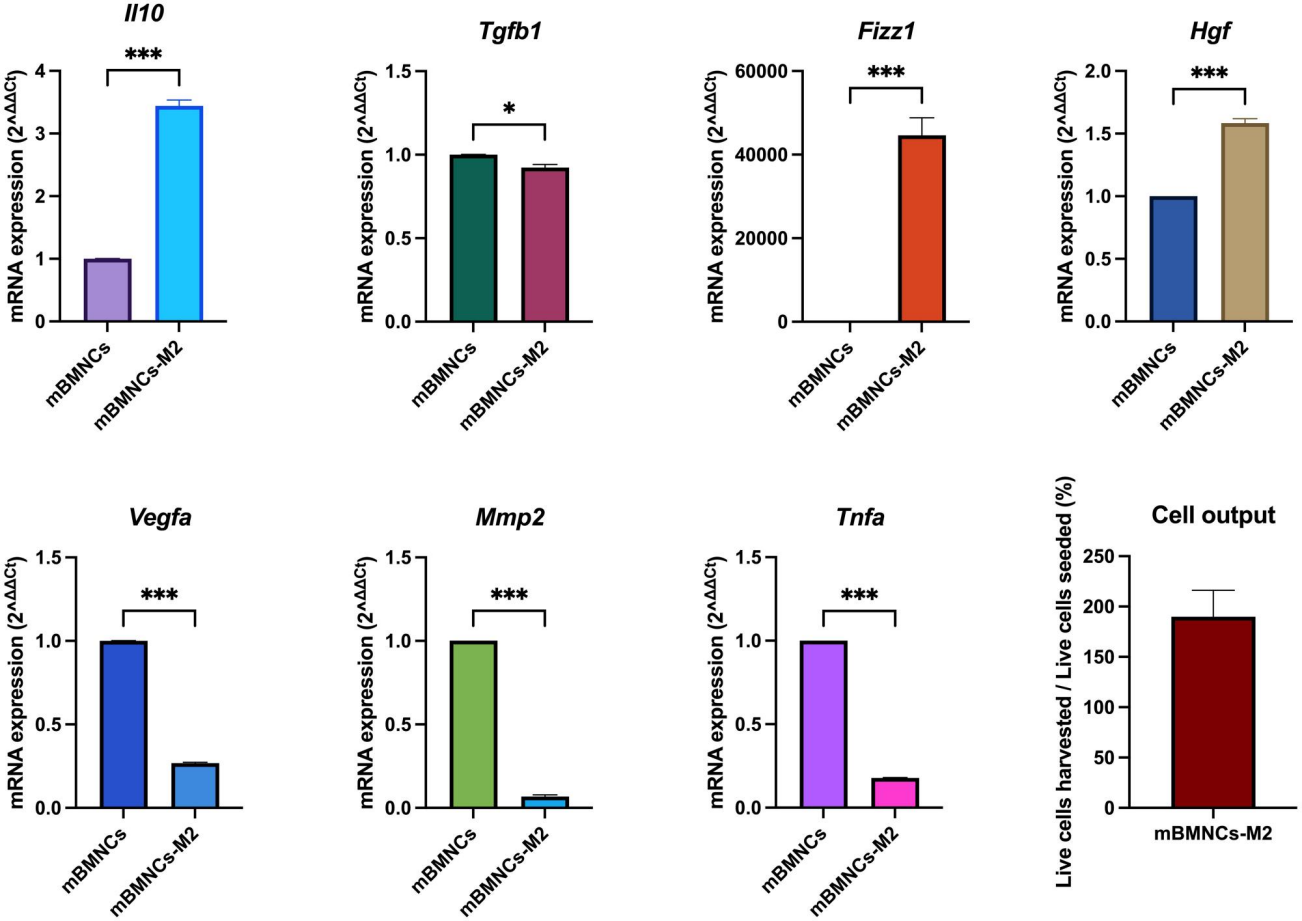
mRNA expression levels of mBMNCs-M2 relative to those of mBMNCs and the cell yield. The RT–qPCR results revealed a reparative phenotype of mBMNCs-M2. The upregulation of *Fizz1, Il10, and Hgf* and decreased levels of *Tnfa*, a potent proinflammatory cytokine, emphasized that mBMNCs-M2 had the ability to heal. The cell yield (ratio of harvested mBMNCs-M2 on day 6 to seeded mBMNCs on day 1) reached 190%. Changes in mRNA levels were assessed by real-time qPCR using *Gapdh* as a reference gene. Histograms are presented as the mean±SEM. The data were obtained from 4 biological samples and 3 technical replicates. *P < 0.05, **P < 0.01, ***P < 0.001. *mBMNCs—mouse bone marrow mononuclear cells freshly isolated from 8–10-week-old male C57BL/6J mice; mBMNCs-M2—mBMNCs-derived M2-like macrophages*

**Figure 6:**
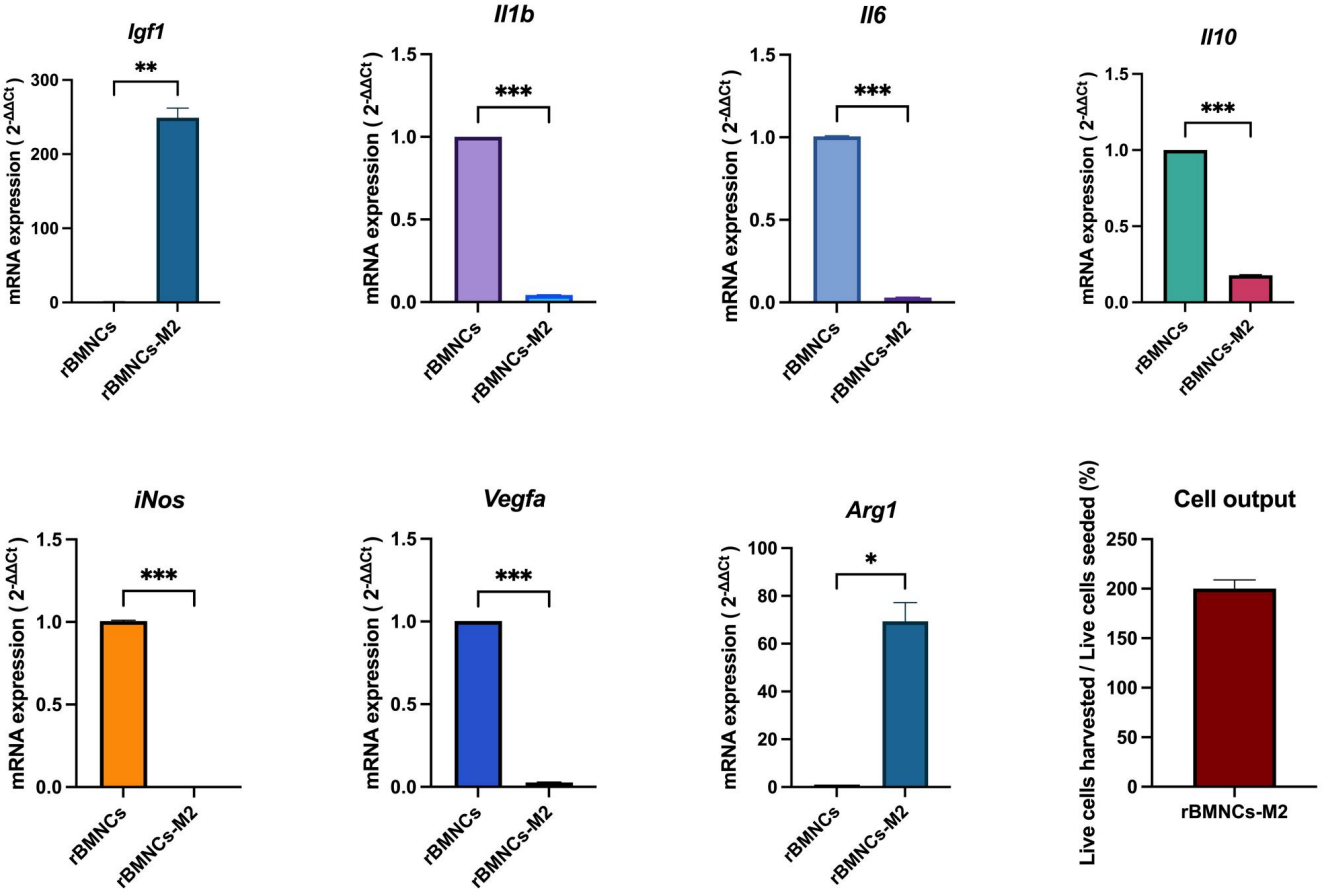
mRNA expression levels of rBMNCs-M2 compared with those of rBMNCs and cell yield. The RT–qPCR results revealed a reparative phenotype of rBMNCs-M2. The overexpression of *Arg1* (a classical marker of the M2 phenotype) and *Igf1* and the downregulation of proinflammatory cytokines, including *Il1b* and *Il6*, as well as the classical identifier of the M1 phenotype, *iNos*, confirmed that rBMNCs-M2 exhibited an M2-like phenotype. The cell yield (the ratio of harvested rBMNCs-M2 on day 6 to seeded rBMNCs on day 1) reached 200%. Changes in mRNA levels were assessed by real-time qPCR using *Gapdh* as a reference gene. Histograms are presented as the mean±SEM. The data were obtained from 4 biological samples and 3 technical replicates. *P < 0.05, **P < 0.01, ***P < 0.001. *rBMNCs—rat bone marrow mononuclear cells freshly isolated from 6–8-week-old male Lewis rats; rBMNCs-M2—rBMNCs-derived M2-like macrophages*

If all steps are performed correctly, the total number of BMNCs collected from each animal, i.e. mice or rats, can reach 1*×*10^7^ and 4*×*10^7^, respectively. Because the initial induction in the first 3 days was based on Mcsf alone, the total number of cells may have decreased during this period (data not shown), and there were still many floating cells (Figs. 3B and 4A). However, supplementation of the cell culture medium with Il4 not only induced Mcsf-treated cells toward the M2 phenotype but also directly or indirectly stimulated cell proliferation, as the cell yield was approximately 200% (Figs. 5 and 6). In addition, from day 4 to day 5 of induction, there was a significant transition in cell morphology. M2-like macrophages had different shapes: some cells were round, whereas others were elongated or spread with protrusions on the membrane. The confluency also increased, and most of the cells attached to the bottom of the plate (Figs. 3B and 4A).

### Reparative properties of both mBMNCs-M2 and rBMNCs-M2

Real-time qPCR of mBMNCs-M2 revealed that M2-like macrophages overexpressed genes encoding *Il10, Fizz1, and Hgf*, whereas the profile of the classical proinflammatory cytokine Tnfa was less highly expressed in the mBMNCs-M2 group than in the freshly isolated mBMNCs (*P* < 0.001) (Fig. 5). The expression of *Fizz1*, a classical marker of M2-like macrophages, was significantly greater in mBMNCs-M2 than in mBMNCs (*P < 0.001*). In addition, *Il10* and *Hgf* were also overexpressed in mBMNCs-M2 (*P < 0.001*). Genes encoding *Vegfa* and *Mmp2* were significantly downregulated in mBMNCs-M2 (*P < 0.001*), while *Tgfb1* mRNA levels in mBMNCs and mBMNCs-M2 were slightly different (*P < 0.05*) (Fig. 5).

A similar trend was observed in the gene profile of rBMNCs-M2 compared with that of rBMNCs: most anti-inflammatory cytokines were overexpressed, and proinflammatory cytokines were downregulated (Fig. 6). The proliferative cytokine Igf1 was upregulated after induction. The only difference between the rBMNC-M2 and mBMNC-M2 groups was in the *Il10* mRNA level compared with that in the respective control groups, where *Il10* expression was upregulated in the mBMNC-M2 group and down-regulated in the rBMNC-M2 group (Figs. 5 and 6). The expression of *Arg1*, another classic marker of M2-like macrophages similar to *Fizz1*, was upregulated in rBMNCs-M2 compared with that in rBMNCs.

### Recognition of M2-like macrophages via flow cytometry

Since there is still no exact consensus on the definition of M2-like macrophages in different studies, the most common phenotypic markers, such as CD11b, CD206, and F4/80 for mBMNCs-M2 and CD68 for rBMNCs-M2, were selected for analysis by flow cytometry. On Day 6, 95.7% of the cells were triple positive for CD11b, CD206, and F4/80. Periodic flow cytometry analysis at 72, 96, 120, 144, and 168 h after induction revealed an increase in F4/80 and CD206 expression by 120 h, with substantial growth from 12.8% (before the medium was changed) to 65.6% (24 h after the medium was changed) and 95.7% (48 h after the medium was changed). This can be explained by the potential of recombinant murine Il4 to induce the M0 phenotype (driven by recombinant murine Mcsf during the first 72 h) toward the M2 phenotype (Fig. 3A). However, the percentages of CD11b-, F4/80-, and CD206-positive cells decreased significantly on Days 7 and 8, at 91.2% and 80.4%, respectively.

Flow cytometry analysis at 72 h after induction or before the medium was changed revealed that 69.4% of the cells expressed CD68 (panmacrophage marker). However, when the CD68 marker was examined on day 6, the expression index reached 99.3% (Fig. 4B).

## Discussion

This study revealed that the use of Mcsf for the first 3 days and the addition of Il4 for the other two days produced CD11b^+^/CD206^+^/F4/80^+^ and *Fizz1*-overexpressing [20] mBMNCs-M2 and CD68^+^ [25] and *Arg1*-overexpressing [26] rBMNCs-M2 cells from mice and rats BMNCs. Overall, the obtained mBMNCs-M2 and rBMNCs-M2 expressed a wide range of reparative and anti-inflammatory cytokines, while the levels of proinflammatory cytokines were reduced. Another advantage of this method of inducing M2-like macrophages is the shorter duration compared with existing protocols [20–23,27–31], where the average induction time exceeds 5 days or 120 h. If the total induction period is approximately 120 h, the actual hands-on time required for procedures such as bone excision, isolation of BMNCs, cell seeding, medium change, and cell harvesting is about 120–150 min per animal. This was achieved by skipping several washing steps [20] and 4-h incubation period of freshly isolated BMNCs at 37°C aimed at removing resident macrophages as reported in previous protocols [21,22]. Although this strategy raises concerns for resident bone marrow-derived macrophages, it allowed us to harvest a broader population of BMNCs, including potential progenitor cells that may contribute to the differentiation of M2-like macrophages. Moreover, flow cytometry analysis assessing the effect of this modification on the purity of harvested cells at day 6 showed that virtually all differentiated cells exhibited the phenotypic cell surface/intracellular markers of M2-like macrophages (Figs. 3A and 4A).

We tested the expression of CD11b^+^/CD206^+^/F4/80^+^ on mBMNCs-M2 at additional two points (144 and 168 h after induction) to determine the most appropriate time for harvesting. The data revealed that the number of CD11b^+^/CD206^+^/F4/80^+^ cells increased gradually toward Days 5 and 6, especially after medium replacement or Il4 supplementation; however, prolonged induction time resulted in a backward profile of the mBMNCs-M2 phenotype (Fig. 3A). With this context, we decided that the most appropriate time for induction is approximately 120 h but not >144 h.

In terms of cell yield, this method can produce more mBMNCs-M2 and rBMNCs-M2 than initially seeded BMNCs. This result could be explained by the effect of Il4 on cell proliferation described in previous studies [32]. To investigate whether Il4 could promote the proliferation of M2-like macrophages by itself, without Mcsf, cells treated with Mcsf alone for the first 72 h were induced with Il4 alone for another two days. Interestingly, Il4 could stimulate cell proliferation only in the presence of Mcsf in the culture medium; otherwise, cell number declined sharply, with a cell yield of approximately 10.2% by day 6. Macrophage survival strongly depends on Mcsf, which activates Ras-Raf-MEK-ERK and PI3K-Akt pathways, activating transcription of anti-apoptotic genes, including pro-survival members of the *Bcl (B-cell lymphoma)* family, phosphorylating and inactivating pro-apoptotic proteins and controlling mitochondrial membrane permeability [33]. Unlike mBMNCs-M2, rBMNCs-M2 once differentiated do not require exogenous Mcsf for survival [17]. Another possible reason for the high cell yield is likely L-ascorbic acid, which was consistently used in this protocol to maintain cell viability. Since the early 1990s, studies have investigated the role of L-ascorbic acid in the immune response against infectious and neoplastic diseases and elucidated its importance in the activation and function of innate and adaptive immune cells [34–36]. One of the putative mechanisms is the inhibition of apoptosis by L-ascorbic acid in human monocytes or a decrease in the secretion of proinflammatory cytokines such as Il6 and Tnfa [37]. However, further studies are needed to understand the exact mechanisms underlying this process, especially when classical macrophage inducers such as Mcsf and Il4 are combined.

Most studies performed on rats face some problems due to the limited availability of reliable and time-tested reagents compared with studies on mice and humans. For this reason, it was not possible to validate all the flow cytometric markers of rBMNCs-M2 in this study. By combining the results of flow cytometry and RT–qPCR performed on rBMNCs-M2, we preferred to define rBMNCs-M2 as CD68-positive and *Arg1*-upregulated cells.

Overall, this method produces M2-like macrophages from murine and rat BMNCs, probably via the same molecular mechanisms. It is clear that there are biological differences between rodents [38,39], which may explain the nonidentical results in terms of the mRNA levels, especially the opposite expression levels of *Il10*. Although direct comparative studies of gene expression profiles between in vitro polarized mBMNCs-M2 and rBMNCs-M2 are limited, existing studies provide insight into species-specific differences in macrophage polarization. For example, Pridans et al (2021) developed a technique to derive macrophages from rat embryonic stem cells (ESCs) and compared their gene expression profiles with macrophages from different tissues and sources of differentiation [17]. Similarly, Lavin et al. (2014) investigated mouse macrophages [40], and a comprehensive review by Orecchioni et al. (2019) considers the interchangeable terminology of M1 versus classically activated and M2 versus alternatively activated macrophages using mouse transcriptome datasets [41]. These and other studies highlight the importance of considering species differences when interpreting macrophage polarization and gene expression data. Therefore, there is no clear consensus on differentiation markers. Most reports and protocols to identify cells polarized *in vitro* or taken from biological tissues as M2-like macrophages use markers that either favor the M2 or M1 phenotype (a relatively indirect strategy that ignores the M1 phenotype, thereby voting for the second option, the M2 phenotype) or both (Table 3) in various combinations, and thus reliable markers as well as combinations thereof have yet to be found. Consistent with this, our data showed the general trend of increased mRNA expression of anti-inflammatory cytokines and decreased expression of proinflammatory cytokines suggesting that mBMNCs-M2 and rBMNCs-M2 may possess potential therapeutic and/or pro-healing efficacy in an *in vivo* assay. The range of molecular mechanisms by which BMNCs acquire the M2 phenotype was beyond the scope of the current study. These questions may be answered in future studies.

**Table 3.** Commonly used phenotypic markers of mouse and rat macrophages.

Species	Markers of monocyte-macrophage transition	Markers associated with M1 polarization	Markers associated with M2 polarization
Mouse	CD11b [20,42,43], CD11c [42–44], F4/80 [20,42–44], CD68 [42]	*Tnfα* [20,42,45], CD80 [42], *iNos* [42,45], CD86 [42], Il12 [45]	CD206 [20,42,44,45], *Arg1* [20,42,45,46], Il10 [42,45], *Fizz1* [20,42,46], *Ym1* [20,42,46]
Rat	CD68 [17,25,47–49]	CD80 [25,50], iNOS [48–50], CCR7 [25], CD86 [47], *Tnfα* [47], IFNγ [47]	CD163 [25,49], CD206 [11,48–50], Il1-R2 [49], CCL17 [49], *Arg1* [47,49], Il10 [47]

## Study limitations

This study has several limitations. First, this study could not analyze the whole spectrum of pro- and anti-inflammatory cytokine profiles; instead, the most commonly used cytokines were tested. Consequently, other cytokines may be expressed differently. Second, all combinations of Mcsf, Il4 and L-ascorbic acid were not analyzed in this study in terms of their concentration and timing, which may influence the observed results. Third, only one strain of mouse (C57Bl/6J) and one strain of rat (Lewis) were used in this work. However, further studies are needed to test whether other strains respond in the same way. Fourth, although the current protocol proved useful for inducing M2-like macrophages in both species, the results, including the expression of cluster differentiation (CD) markers, mRNA levels, and definitions of M2-like macrophages, were not identical due to possible interspecies biological differences. Finally, the potential therapeutic efficacy and safety of mBMNCs-M2 and rBMNCs-M2 were not tested in this study and should be investigated in future studies from different perspectives using both small and large animal disease models, as the current study was aimed primarily at developing a straightforward and efficient way to induce M2-like macrophages from BMNCs rather than focusing on *in vivo* efficacy and safety.

## Conclusion

The current protocol for polarization of M2-like macrophages is a simple, reproducible, time- and cost-effective approach with high cell yields. Therefore, this method can serve as a valuable tool for macrophage research. To obtain high-quality M2-like macrophages, strict sterility and proper handling of cytokines must be ensured.

## Data Availability

The data underlying this study will be made available upon reasonable request to the corresponding author.

## References

[1] Hume DA. The many alternative faces of macrophage activation. Front Immunol 2015;6:370. 10.3389/fimmu.2015.00370 26257737 PMC4510422

[2] Hume DA , Mabbott N, Raza S et al Can DCs be distinguished from macrophages by molecular signatures? Nat Immunol 2013;14:187–9. 10.1038/ni.2516 23416664

[3] Mosser DM. The many faces of macrophage activation. J Leukoc Biol 2003;73:209–12. 10.1189/jlb.0602325 12554797

[4] Mosser DM , Edwards JP. Exploring the full spectrum of macrophage activation. Nat Rev Immunol 2008;8:958–69. 10.1038/nri2448 19029990 PMC2724991

[5] Lampiasi N. Macrophage Polarization: learning to Manage It 2.0. Int J Mol Sci 2023;24:17409. 10.3390/ijms242417409 PMC10743686 38139238

[6] Liang Y , Xu Q, Gao Q. Advancing CAR-based immunotherapies in solid tumors: CAR-macrophages and neutrophils. Front Immunol 2023;14:1291619. 10.3389/fimmu.2023.1291619 38090576 PMC10715261

[7] Hadiloo K , Taremi S, Heidari M et al The CAR macrophage cells, a novel generation of chimeric antigen-based approach against solid tumors. Biomark Res 2023;11:103. 10.1186/s40364-023-00537-x PMC10685521 38017494

[8] Dziki JL , Wang DS, Pineda C et al Solubilized extracellular matrix bioscaffolds derived from diverse source tissues differentially influence macrophage phenotype. J Biomed Mater Res A 2017;105:138–47. 10.1002/jbm.a.35894 27601305

[9] Barnett-Vanes A , Sharrock A, Birrell MA et al A single 9-colour flow cytometric method to characterise major leukocyte populations in the rat: validation in a model of LPS-induced pulmonary inflammation. PLoS One 2016;11:e0142520. 10.1371/journal.pone.0142520 26764486 PMC4713146

[10] Kozłowski HM , Sobocińska J, Jędrzejewski T et al Fever-range hyperthermia promotes macrophage polarization towards regulatory phenotype M2b. Int J Mol Sci 2023;24:17574. 10.3390/ijms242417574 PMC10744093 38139402

[11] Xun WL , Xi ZS, Juan WH et al M2b macrophage polarization and its roles in diseases. J Leukoc Biol 2019;106:345–58. 10.1002/JLB.3RU1018-378RR 30576000 PMC7379745

[12] Ruiz-Torres SJ , Bourn JR, Benight NM et al Macrophage-mediated RON signaling supports breast cancer growth and progression through modulation of IL-35. Oncogene 2022;41:321–33. 10.1038/s41388-021-02091-y 34743208 PMC8758553

[13] Liu Y , Wang J, Zhang J et al Targeting tumor-associated macrophages by MMP2-sensitive apoptotic body-mimicking nanoparticles. ACS Appl Mater Interfaces 2020;12:52402–14. 10.1021/acsami.0c15983 33169982 PMC8229024

[14] Moore KJ , Freeman MW. Embryonal stem (ES) cell-derived macrophages: a cellular system that facilitates the genetic dissection of macrophage function. Vascular Dis 2003;30:343–55. 10.1385/1-59259-247-3:343 21341038

[15] Lindmark H , Rosengren B, Hurt-Camejo E et al Gene expression profiling shows that macrophages derived from mouse embryonic stem cells is an improved in vitro model for studies of vascular disease. Exp Cell Res 2004;300:335–44. 10.1016/j.yexcr.2004.06.025 15474998

[16] Odegaard JI , Vats D, Zhang L et al Quantitative expansion of ES cell-derived myeloid progenitors capable of differentiating into macrophages. J Leukoc Biol 2007;81:711–9. 10.1189/jlb.0906590 17158607 PMC1904487

[17] Pridans C , Irvine KM, Davis GM et al Transcriptomic analysis of rat macrophages. Front Immunol 2021;11:594594. 10.3389/fimmu.2020.594594 33633725 PMC7902030

[18] Pouyanfard S , Meshgin N, Cruz LS et al Human induced pluripotent stem cell-derived macrophages ameliorate liver fibrosis. Stem Cells 2021;39:1701–17. 10.1002/stem.3449 34460131

[19] Senju S , Haruta M, Matsunaga Y et al Characterization of dendritic cells and macrophages generated by directed differentiation from mouse induced pluripotent stem cells. Stem Cells 2009;27:1021–31. 10.1002/stem.33 19415766

[20] Podaru MN , Fields L, Kainuma S et al Reparative macrophage transplantation for myocardial repair: a refinement of bone marrow mononuclear cell-based therapy. Basic Res Cardiol 2019;114:34. 10.1007/s00395-019-0742-1 31372765 PMC6675756

[21] Trouplin V , Boucherit N, Gorvel L et al Bone marrow-derived macrophage production. J Vis Exp 2013;e50966. 10.3791/50966 24300014 PMC3991821

[22] Weisser SB , McLarren KW, Kuroda E et al Generation and characterization of murine alternatively activated macrophages. Methods Mol Biol 2013;946:225–39. 10.1007/978-1-62703-128-8_14 23179835

[23] Ma SF , Chen YJ, Zhang JX et al Adoptive transfer of M2 macrophages promotes locomotor recovery in adult rats after spinal cord injury. Brain Behav Immun 2015;45:157–70. 10.1016/j.bbi.2014.11.007 25476600

[24] Useful Numbers for Cell Culture | Thermo Fisher Scientific—JP. Accessed February 9, 2025. https://www.thermofisher.com/jp/ja/home/references/gibco-cell-culture-basics/cell-culture-protocols/cell-culture-useful-numbers.html

[25] Badylak SF , Valentin JE, Ravindra AK et al Macrophage phenotype as a determinant of biologic scaffold remodeling. Tissue Eng Part A 2008;14:1835–42. 10.1089/ten.tea.2007.0264 18950271

[26] Yang Z , Ming XF. Functions of arginase isoforms in macrophage inflammatory responses: impact on cardiovascular diseases and metabolic disorders. Front Immunol 2014;5:533. 10.3389/fimmu.2014.00533 25386179 PMC4209887

[27] Guo XY , Wang SN, Wu Y et al Transcriptome profile of rat genes in bone marrow-derived macrophages at different activation statuses by RNA-sequencing. Genomics 2019;111:986–96. 10.1016/j.ygeno.2018.06.006 31307632

[28] Spiller KL , Wrona EA, Romero-Torres S et al Differential gene expression in human, murine, and cell line-derived macrophages upon polarization. Exp Cell Res 2016;347:1. 10.1016/j.yexcr.2015.10.017 26500109

[29] Lee S , Huen S, Nishio H et al Distinct macrophage phenotypes contribute to kidney injury and repair. J Am Soc Nephrol 2011;22:317–26. 10.1681/ASN.2009060615 21289217 PMC3029904

[30] Enderlin Vaz Da Silva Z , Lehr HA, Velin D. In vitro and in vivo repair activities of undifferentiated and classically and alternatively activated macrophages. Pathobiology 2014;81:86–93. 10.1159/000357306 24457836

[31] Cao Q , Wang Y, Zheng D et al Failed renoprotection by alternatively activated bone marrow macrophages is due to a proliferation-dependent phenotype switch in vivo. Kidney Int 2014;85:794–806. 10.1038/ki.2013.341 24048378

[32] Jenkins SJ , Ruckerl D, Thomas GD et al IL-4 directly signals tissue-resident macrophages to proliferate beyond homeostatic levels controlled by CSF-1. J Exp Med 2013;210:2477–91. 10.1084/jem.20121999 24101381 PMC3804948

[33] Sester DP , Brion K, Trieu A et al CpG DNA activates survival in murine macrophages through TLR9 and the phosphatidylinositol 3-Kinase-Akt pathway. J Immunol 2006;177:4473–80. 10.4049/jimmunol.177.7.4473 16982883

[34] Abobaker A , Alzwi A, Alraied AHA. Overview of the possible role of vitamin C in management of COVID-19. Pharmacol Rep 2020;72:1517–28. 10.1007/s43440-020-00176-1 33113146 PMC7592143

[35] Carr AC , Maggini S. Vitamin C and immune function. Nutrients 2017;9:1211. 10.3390/nu9111211 PMC5707683 29099763

[36] Brabson JP , Leesang T, Mohammad S et al Epigenetic regulation of genomic stability by vitamin C. Front Genet 2021;12:675780. 10.3389/fgene.2021.675780 34017357 PMC8129186

[37] Mousavi S , Bereswill S, Heimesaat MM. Immunomodulatory and antimicrobial effects of vitamin C. Eur J Microbiol Immunol (Bp) 2019;9:73–9. 10.1556/1886.2019.00016 31662885 PMC6798581

[38] Cunningham ML. A mouse is not a rat is not a human: species differences exist. Toxicol Sci 2002;70:157–8. 10.1093/toxsci/70.2.157 12441359

[39] Khalaf AA , Muhsan AZ. Biological Comparison Between Mice and Rats. Clin Res Anim Sci 2022;3:000537. 10.31031/CRAS.2022.02.000537

[40] Lavin Y , Winter D, Blecher-Gonen R et al Tissue-resident macrophage enhancer landscapes are shaped by the local microenvironment. Cell 2014;159:1312–26. 10.1016/j.cell.2014.11.018 25480296 PMC4437213

[41] Orecchioni M , Ghosheh Y, Pramod AB et al Macrophage polarization: different gene signatures in M1(Lps+) vs. Classically and M2(LPS-) vs. Alternatively activated macrophages. Front Immunol 2019;11:234. 10.3389/fimmu.2019.01084 PMC6543837 31178859

[42] A Guide to Macrophage Markers | Biocompare: The Buyer’s Guide for Life Scientists. Accessed February 11, 2025. https://www.biocompare.com/Editorial-Articles/566347-A-Guide-to-Macrophage-Markers/? utm_source=chatgpt.com

[43] Arumugam P , Suzuki T, Shima K et al Long-term safety and efficacy of gene-pulmonary macrophage transplantation therapy of PAP in Csf2ra−/− mice. Mol Ther 2019;27:1597–611. 10.1016/j.ymthe.2019.06.010 31326401 PMC6731469

[44] Mao R , Wang C, Zhang F et al Peritoneal M2 macrophage transplantation as a potential cell therapy for enhancing renal repair in acute kidney injury. J Cell Mol Med 2020;24:3314–27. 10.1111/jcmm.15005 32004417 PMC7131941

[45] Li K , Xu W, Guo Q et al Differential macrophage polarization in male and female BALB/c mice infected with coxsackievirus B3 defines susceptibility to viral myocarditis. Circ Res 2009;105:353–64. 10.1161/CIRCRESAHA.109.195230 19608981

[46] Liu H , He Y, Lu C et al Efficacy of pulmonary transplantation of engineered macrophages secreting IL-4 on acute lung injury in C57BL/6J mice. Cell Death Dis 2019;10:664. 10.1038/s41419-019-1900-y 31511535 PMC6739369

[47] Martín-Fernández B , Rubio-Navarro A, Cortegano I et al Aldosterone induces renal fibrosis and inflammatory M1-macrophage subtype via mineralocorticoid receptor in rats. PLoS One 2016;11:e0145946. 10.1371/journal.pone.0145946 26730742 PMC4701403

[48] Grotenhuis N , Vd Toom HFE, Kops N et al In vitro model to study the biomaterial-dependent reaction of macrophages in an inflammatory environment. Br J Surg 2014;101:983–92. 10.1002/bjs.9523 24838620

[49] Zhang XL , Guo YF, Song ZX et al Vitamin D prevents podocyte injury via regulation of macrophage M1/M2 phenotype in diabetic nephropathy rats. Endocrinology (United States) 2014;155:4939–50. 10.1210/en.2014-1020 25188527

[50] Palmer JA , Abberton KM, Mitchell GM et al Macrophage phenotype in response to implanted synthetic scaffolds: an immunohistochemical study in the rat. Cells Tissues Organs 2014;199:169–83. 10.1159/000363693 25412799

